# Implementation of a shared medication list in primary care – a controlled pre-post study of medication discrepancies

**DOI:** 10.1186/s12913-021-07346-8

**Published:** 2021-12-13

**Authors:** Anette Vik Josendal, Trine Strand Bergmo, Anne Gerd Granas

**Affiliations:** 1 Norwegian Centre for E-health Research, University Hospital of North Norway, Tromsø, Norway; 2 Department of Pharmacy, University of Oslo, Oslo, Norway; 3 Department of Pharmacy, UiT The Arctic University of Norway, Tromsø, Norway

**Keywords:** Shared medication list, Multidose drug dispensing, Medication reconciliation, Medication discrepancies, Primary care, E-health, E-medicines management

## Abstract

**Background:**

Access to medicines information is important when treating patients, yet discrepancies in medication records are common. Many countries are developing shared medication lists across health care providers. These systems can improve information sharing, but little is known about how they affect the need for medication reconciliation. The aim of this study was to investigate whether an electronically Shared Medication List (eSML) reduced discrepancies between medication lists in primary care.

**Methods:**

In 2018, eSML was tested for patients in home care who received multidose drug dispensing (MDD) in Oslo, Norway. We followed this transition from the current paper-based medication list to an eSML. Medication lists from the GP, home care service and community pharmacy were compared 3 months before the implementation and 18 months after. MDD patients in a neighbouring district in Oslo served as a control group.

**Results:**

One hundred eighty-nine patients were included (100 intervention; 89 control). Discrepancies were reduced from 389 to 122 (*p* <  0.001) in the intervention group, and from 521 to 503 in the control group (*p* = 0.734). After the implementation, the share of mutual prescription items increased from 77 to 94%. Missing prescriptions for psycholeptics, analgesics and dietary supplements was reduced the most.

**Conclusions:**

The eSML greatly decreases discrepancies between the GP, home care and pharmacy medication lists, but does not eliminate the need for medication reconciliation.

## Background

Access to medicines information is important when treating patients. If treatment decisions are based on outdated medication lists this can lead to inappropriate prescribing, discontinuity of therapy and medication errors [1, 2]. Yet, discrepancies in medication lists are common, particularly during transitions of care [3]. A systematic review has shown that up to 60–67% of medication histories recorded at hospital admissions contain at least one medication discrepancy, 11–59% of these were clinically important [2]. These discrepancies may not only result in inappropriate treatment during the hospital stay but also carry over to discharge, resulting in errors in the discharge letters to primary care providers [4, 5].

Also within primary care, discrepancies are common and studies show that up to 90% of patients have at least one discrepancy in their lists [6–8]. Poor communication between health care providers is a common cause of these discrepancies [1, 9–13]. In addition, there are many manual routines involved in the transfer of medicines information [8, 14, 15]. A recent Norwegian study shows that primary care nurses, pharmacists and GPs experience many challenges obtaining an accurate medication list. They find the current procedures very time consuming, complex and posing a risk to patient safety [15]. Technology such as e-prescribing, which can increase legibility and completeness of the prescriptions and increase access to medicines information, has been suggested to address these challenges [16, 17]. In addition, several countries are developing systems for sharing complete medication lists across care levels [18, 19]. These systems for sharing medication lists vary between countries, and the scientific evidence on the effects is still limited [19].

In Norway, e-prescribing in primary care was implemented in 2013, and today more than 90% of new prescriptions are sent electronically [20]. The Norwegian Directorate of eHealth is also currently developing a nationwide electronic Shared Medication List (eSML) [21]. The first patients to get an eSML are home care service patients with multidose drug dispensing (MDD), a system where patients get medicines dispensed as unit-for-use disposable bags. Today, MDD patients already have a complete medication list containing all regular medications, *when needed* medications and dietary supplements, but this list is paper-based and sent by fax between the actors. Though one Norwegian study has shown that the paper-based MDD system can reduce the number of discrepancies in medication lists [7], discrepancies between the community pharmacy, the home care services and the GPs still frequently occur in the paper-based system [7, 22, 23]. When eSML is implemented for these patients, the paper-based medication list will be replaced by a joint electronic list. In addition, e-prescriptions for each item on the eSML is necessary to dispense medicines. Both the e-prescriptions and the eSML are transferred via a national database accessible from all pharmacies and prescribers in the country.

The goal of the eSML is to generate one structured and complete medication list, to increase access to medicines information for all health care professionals involved in the care of MDD patients and to reduce the time used on medication reconciliation [21, 24]. In this study, we investigate whether the eSML system decreases the number of discrepancies between the medication lists of the GP, home care service and the community pharmacy.

## Methods

### Study setting

This study followed the implementation of the eSML for MDD patients in Oslo in 2018. The Directorate of eHealth was responsible for the implementation process and chose participants based on the GPs electronic health record (EHR) system. All GPs with a specific EHR system in a given district in Oslo were asked to participate.

### Study design and sample

This study has a controlled pre-post design. The Directorate of eHealth provided contact details for the health care personnel who were starting the eSML in Oslo: 3 GP offices with a total of 17 GPs, one home care service district and one community pharmacy. GP offices located in the same or a neighbouring district in Oslo who were not using eSML served as a control group. GPs were recruited until we had the same number of estimated patients in the control group as in the intervention group. The home care services in the control district was also contacted. The same pharmacy provided MDD to both districts.

### The intervention

Before the intervention, all GPs used paper-based MDD-prescriptions, mostly printouts from the medication list in the GPs Electronic Health Record (EHR). These prescriptions are usually complete medication lists containing all regular medications, *when needed* medications, medical devices and dietary supplements, and are valid for 1 year supply of all items on the list. The GP sends the MDD prescription to the pharmacy via fax. The hospital doctors can also prescribe for these patients, but the main rule is that the GP approve these changes before they are dispensed in MDD. If the prescribing is done via ordinary electronic prescriptions, the MDD pharmacy will not automatically be notified about the prescription.

The eSML prescribing system is a function in the currently used EHR systems. After this functionality is turned on, the GP can define which patients should use eSML. The eSML by itself is only a medication list giving an overview of the patient’s current treatment, but it cannot be used for dispensing directly. It is thus necessary to generate e-prescriptions for each item on the eSML. When a patient is defined as using eSML in the EHR system, the eSML will be generated and sent automatically when the GP generate e-prescriptions. Both the eSML and the e-prescriptions are valid for 1 year, however, the e-prescriptions also contain information about the quantity which can be dispensed on the prescription. This means that the e-prescriptions can be emptied before 1 year has passed. At present, all prescribers have access to read the eSML, but only the GPs can update the list [24]. When hospital physicians prescribe medicine, they will do so by ordinary e-prescriptions. They should also withdraw prescriptions that are no longer relevant or appropriate. As in the paper-based system, the current recommendation is that the pharmacy should wait to dispense MDD until the GP has updated the eSML, but it is possible to dispense MDD on the e-prescriptions if the pharmacist deems it necessary. For MDD patients, the system also opens for electronic communication between the GP and the MDD dispensing pharmacy, where the pharmacist can suggest changes of the eSML directly to the GP. If the pharmacist dispenses a prescription that is not included in the eSML, e.g. a prescription from the hospital or a dietary supplement, the system will automatically send information about this to the GP.

During the first pilot testing of the eSML system in 2014, the nurses and pharmacists experienced many errors in the first eSML created. They retrospectively reported errors to the GPs asking them to correct the lists [23, 25, 26]. It was suspected that these errors were caused by the discrepancies in the medication lists. In our study, the Directorate of eHealth thus recommended medication reconciliation of the medication lists at the GP, pharmacy and/or home care before the actual creation of the eSML. To facilitate this, the MDD pharmacy sent a printout of their medication lists to the GPs approximately 1 month before start-up. After comparing this list to their own record, the GP created the first eSML and necessary e-prescriptions. The GPs could claim reimbursement for this work if they documented it as a medication review, a process that all GPs in Norway can be reimbursed for up to three times a year for patients with four or more medications [27]. Once the pharmacy received the eSML for the patients, they deleted the paper-based medication list in their system and started dispensing MDD based on the new eSML and e-prescriptions.

### Data collection

A list of all MDD patients in the two districts who were registered with one of the participating GPs was compiled using the pharmacy dispensing programme. One week before data collection, a letter was sent to all participants (all GPs, the home care services and the pharmacy) with the list of MDD patients under their care and a generated serial number for each patient. The following week, the participants printed out the medication lists for the patients and replaced the patient identifying information with the serial number. The lists were posted or collected in person by AVJ. For the intervention group, the first medication lists were collected in March 2018, approximately 3 months prior to the implementation of eSML. For the control group, the first medication lists were collected in June 2018. For both groups, the lists were collected again in September and October 2019.

### Analyses

The medication lists were compared in pairs: GP-list – Home-care-list; GP-list – Pharmacy-list; Pharmacy-list – Home-care-services-list. Two researchers separately compared each set. First, the number of unique prescription items in each list were recorded into four groups: 1) regular prescription items dispensed as MDD; 2) regular prescription items not dispensed as MDD; 3) medications prescribed to be used as required; 4) medical devices and consumables (e.g., diabetes supplies; incontinence products). A unique prescription item was defined by an ATC code for medicines [28], an active ingredient for dietary supplements and a product group for medical devices and consumables [29]. Medicines listed as ‘courses’ in the GP journal system (e.g., short antibiotic courses) were excluded. Second, based on a previous classification system [7, 22, 23], discrepancies were classified in the following categories: Medication lacking from one of the two lists; different dosage; prescriptions written as ‘regular use’ in one list and ‘as required’ in the other; different administration formula; others (see Table 2). Both missing and discordant information in the medication lists were recorded. Lastly, all three lists for the patient were compared to register the number of unique prescription items per patient and the number of prescription items present in all three lists (mutual prescription items). These were used to calculate the congruence level = mutual prescriptions items/unique prescription items.

The three-way comparison of medication lists was only used for the overall congruence level. For the rest of the results, the number of discrepancies between the GP list and the home care service list was used. This was chosen because these were the lists most frequently compared in previous studies. Similarly, the number of items in the GP medication list was used when discussing the number of items prescribed.

Data was registered in Microsoft Office Excel 2016 and analyses were performed in Stata/MP 16.1. The student's t-test was used for continous data to test the significance of differences between groups and changes in time, a chi-square test was used to compare categorical data and McNemar test for paired nominal data. The significance level was set to 0.05. To estimate the effect of the eSML on discrepancies a difference in difference (DID) method was used. The DID design is based on taking the difference in discrepancies before and after the introduction of the eSML, minus the corresponding change in the control group. The main assumption of this method is that of “parallel trends”: that the development of discrepancies would be the same in the two groups in absence of the intervention. Because the method looks at change and not absolute values, the groups can have different baseline levels of the outcome. It also implies that any time-varying factor, such as an information campaign to make GPs reconcile their medication lists, would affect both groups equally and thus not confound the results.

### Ethics

This study was performed in accordance with guidelines and regulations as stated in the study protocol approved by the Data Protection Officer at the University Hospital of North Norway (UNN) (Project No. 02003). Because the aim of the project was not to generate new knowledge about health or disease, but rather quality assurance of a new system, the project fell outside the scope of the Health Research Act. The Regional Committee for Medical Research Ethics (REK) has waived the need to obtain consent for the collection and analyses of the medication lists in this study, due to difficulties contacting home care service patients and an anticipated high dropout rate in this population (57% dropout in a similar study of multidose patients [7]) (2017/1393/REK Nord). Patient identifying data was stored separately from the anonymous medication list in a secure research server at UNN.

## Results

In the intervention group, all 17 GPs who piloted the eSML participated. The data collection before implementation included complete sets of medication lists for 188 patients, the second 100 sets (53%). Of the dropouts, 82 were no longer eligible (moved to another municipality, moved to a nursing home, changed their GP or stopped using MDD); 6 still used MDD but were not available (missing or incomplete medication list from either home care service, GP or pharmacy). Of 12 GP offices contacted for the control group, five offices with 19 GPs accepted the invitation. The reason for declining was lack of time. The first data collection included complete sets for 178 patients, the second 89 (50%). Of the dropouts, 68 were no longer eligible, 21 medication lists were not available.

### Comparison of intervention and control group before implementation

Table 1 shows the comparison of groups before the intervention; the participants in the control group were significantly older than those in the intervention group, but there was not a significant difference in gender or number of items prescribed.

**Table 1:** Pre-intervention comparison of age, gender and number of drugs

Parameter	Intervention, ***N*** = 100	Control, ***N*** = 89	***P***-value*
Age, mean (SD)	63.1 (20.6)	72.5 (19.2)	< 0.001
Gender Female, n (%)	52 (52)	53 (60)	= 0.297
Number of drugs, mean (SD)	10.5 (6.8)	9.4 (5.6)	= 0.891

*p-values calculated with the use of a Chi-square test of independence and Student’s t-test

**Fig. 1:**
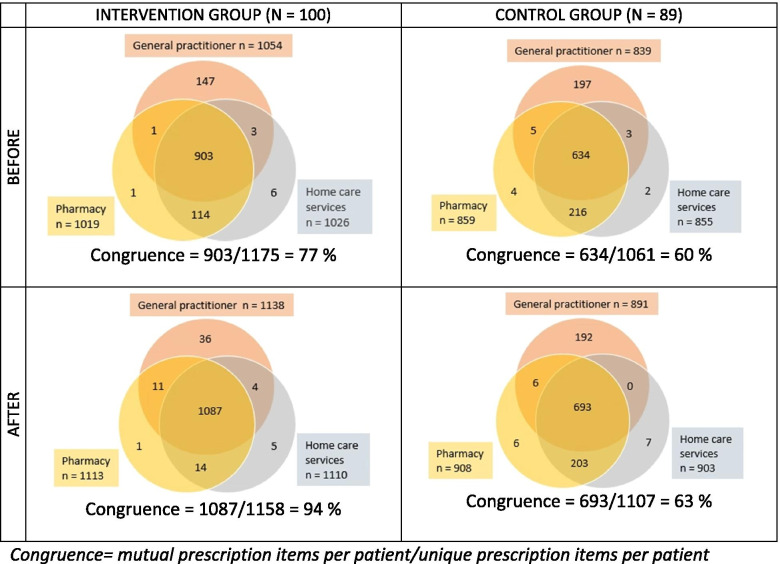
Overlap of medicines information between lists at the GP, home care service and the pharmacy, before and after implementation of an electronic Shared Medication List. Congruence = mutual prescription items per patient/unique prescription items per patient

As seen in Fig. 1, the overlap of medicines information between the three lists was higher in the intervention group than in the control group before the intervention. The intervention group had on average 3.9 discrepancies in their medication lists before the intervention, while the control group had 5.9 (Table 2).

**Table 2:** Type and frequency of discrepancies between the GP and home care services medication list, before and after the implementation of eSML for MDD patients

	INTERVENTION GROUP (***N*** = 100)			CONTROL GROUP (***N*** = 89)		
**NUMBER OF DISCREPANCIES**	**BEFORE**	**AFTER**	Mean difference (95%CI) *p*-value**	**BEFORE**	**AFTER**	Mean difference (95%CI) *p*-value**
Type of discrepancy	n (%)	n (%)		n (%)	n (%)	
Missing prescription	268 (69)	66 (54)		420 (81)	408 (81)	
Dosage	59 (15)	48 (39)		71 (14)	72 (14)	
Regular vs. as required*	50 (13)	5 (4)		18 (3)	19 (4)	
Pharmaceutical form	6 (2)	1 (1)		8 (2)	4 (1)	
Other	6 (2)	2 (2)		4 (1)	0 (0)	
Total	389 (100)	122 (100)	−2.6 (−3.57, −1.63)	521 (100)	503 (100)	−0.16 (−0.76, 0.07)
			*p* < 0.001			*p* = 0.734
**NUMBER OF PATIENTS WITH DISCREPANCIES**			*P*-value***			*P*-value***
Missing prescription	60 (60)	38 (38)		69 (78)	73 (82)	
Dosage	36 (36)	27 (27)		45 (51)	45 (51)	
Regular vs. as required*	30 (30)	5 (5)		17 (19)	17 (19)	
Pharmaceutical form	6 (6)	1 (1)		8 (9)	3 (3)	
Other	5 (5)	1 (1)		3 (3)	0 (0)	
Total	75 (75)	56 (56)	0.006	80 (90)	79 (89)	0.782

^*^medicine listed as ‘regular use’ in one list and ‘as required’ in the other

^**^paired t-test

^***^McNemar test

For both groups, when comparing the GP and the home care service list, the most frequent types of discrepancies were that medication was lacking or that different dosages were listed. The most frequent items lacking from the lists were N05-Psycholeptics (11% of missing items before implementation), N02 – Analgesics (10%), dietary supplements (9%), and medical devices and consumables (6%). Medicines acting on the cardiovascular system (ATC-code = C) constituted 10% of the discrepancies.

### Changes after the intervention

Discrepancies in the intervention group were significantly reduced after the implementation of eSML. The share of mutual prescription items in all three lists increased from 77 to 94% (Fig. 1), and the total number of discrepancies was reduced from 383 to 122 (*p* < 0.001) (Table 2). The most frequent types of discrepancies were still that medication was lacking and that different dosages were listed.

After implementation, medical devices and consumables constituted 30 of the 47 missing prescription items in the home care service list. Dietary supplements, which was the most frequently missing item from the GP list before implementation (16% of missing prescription items in the GP list), were not missing from any medication lists after implementation.

No significant reduction in the total number of discrepancies was found in the control group. The share of mutual prescription items in all three lists increased from 60 to 63% (Fig. 1), and the total number of discrepancies was reduced from 517 to 503, but the reduction was not significant (*p* = 0.734) (Table 2). Like in the first data collection, the most frequent types of discrepancies in all list pairs were that a medication was lacking and that a different dosage were listed.

The DID estimation (Δintervention - Δcontrol) found the decrease in number of discrepancies attributable to the eSML to be − 2.44 (− 3.70, − 1.12), *p* < 0.001.

As seen in Fig. 1 there is an increase in the number of items in all medication lists post-intervention. We analyzed the change in the number of prescribed items post hoc and found an increase of 0.25 in the intervention group relative to the control group, however, this was not statistically significant (*p* = 0.54).

## Discussion

This study shows that introduction of a shared medication list significantly reduces the number of discrepancies between the medication lists of the GP, pharmacy, and home care service: the number of discrepancies was reduced by two thirds and the share of patients with discrepancies in their lists decreased from 75 to 56%. The study thus adds to the existing evidence that e-prescribing has the potential to reduce medication discrepancies and prescription errors. It also adds to the limited evidence about discrepancies in the home care setting [30]. Though our study is one of the first to investigate the effect of an eSML on discrepancies specifically [31], e-prescribing is known to increase the legibility, completeness and clarity of prescriptions [32–35], all of which one would also expect to reduce discrepancies.

### Limitations

This study has some important limitations to be aware of when interpreting the results. When the eSML was implemented, the GPs in the intervention groups were recommended to do a medication reconciliation. No such recommendation was given to the GPs enrolled in the control group. It is thus difficult to separate the effect of the reconciliation from that of having an eSML. However, previous studies have shown that even after reconciliations, discrepancies remain or quickly arise again, and reconciliations need to be repeated regularly to keep the medication lists updated [2,36]. After 16 months, the effects of the single medication reconciliation that was done at implementation would probably have dissipated. It is thus likely that the decreased number of discrepancies is mostly due to how the eSML system supports the processes of keeping medication lists updated.

There were some differences between the control group and the intervention group before the implementation. A reason might be that the GPs started to prepare and perform a medication reconciliation before the implementation of eSML [23]. Also, the GPs who accepted to test the eSML may be more positive towards new technology. If this is the case, the effects of eSML on discrepancies might be larger than our results indicate.

The main assumption of a DID analysis is that of parallel trends, i.e., that the trend in the number of discrepancies would be the same for the intervention group in absence of the intervention as the trend in the control group. With this assumption, the differences in the groups at baseline do not necessarily confound the results, given that these differences represent a permanent difference between the two groups. If, however, the differences at baseline are related to e.g. the GPs in the intervention group having better routines for updating the medication lists in the paper-based system, the parallel trend assumption would be violated. Because we only had one data collection before the intervention, we could not test if the parallel trends assumption holds. There is thus uncertainty concerning the magnitude of the change in discrepancies related to the intervention. Another limitation is that this is a relatively small study with patients from only two districts in one municipality, and generalizations should thus be done with caution.

### The effect of eSML on discrepancies

Before the implementation of eSML, the patients had on average 3.9 discrepancies in their medication lists. In line with previous studies, cardiovascular agents, sedatives and analgesics, were among the medications most frequently involved in discrepancies [2]. Cardiovascular medicines and analgesics medicines are also frequently involved in adverse drug events [37–39]. Though reducing these discrepancies could reduce the potential harm, systematic reviews have shown conflicting results on outcomes such as re-hospitalizations and deaths [3, 40, 41]. However, considering the amount of time health care personnel use on medication reconciliation, a more accessible and correct medication list will probably reduce the workload related to these activities.

Much of the reduction in discrepancies were related to items of less clinical importance, such as dietary supplements and medical devices. Since many dietary supplements are dispensed in MDD, the patients will not get these dispensed if they are not listed in the eSLM. For the medical devices and consumables, the patients need prescriptions on these items to get them reimbursed. If these items are missing from the medication lists it might thus have economic consequences for the patients. After the implementation of eSML, one-third of missing prescription items in the home-care service list were related to “medical devices and consumables”. The great increase of this discrepancy type indicates a systematic error in the registration of these in the home care service list after the implementation. This error is probably related to the home care service not having access to the eSML in the prescription database. If this is the case, this problem will be resolved in time for further implementation of the eSML [21].

After implementing eSML, 56% of the patients still have one or more discrepancies in their list (Table 2). There are several potential reasons for these discrepancies. The pharmacists who piloted the eSML experienced that they had to intervene on the prescriptions more frequently in the new system, mostly because the GPs had prescribed an outdated item number or the wrong quantity of medication [42]. This is in line with previous studies showing that e-prescriptions increase the need for pharmacist interventions [43–45]. Such manual changes increase the chance of discrepancies in the medication lists. Another reason might be the delay of the discharge summary from the hospitals [11]. The GP will typically wait for the discharge summary before making changes to their medication list [46], the home care service and the pharmacy, however, will add medications to their lists based on the electronic nursing discharge notes from the hospital and new prescriptions in the national prescription database.

### Implications and further studies

Our study emphasizes the need to do a medication reconciliation and review before the implementation of the eSML, a need also expressed by the GPs who participated in the testing of the system [25]. In the paper-based system, the medications are dispensed based on the medication list in the pharmacy, while after the implementation, the eSML generated by the GP is used for dispensing. Our study found discrepancies in 75–90% of these medication lists. The transition from the paper-based to the electronic system can thus lead to unintended changes in the patient’s medication treatment if these discrepancies are not resolved before implementation.

To avoid errors in the eSML it is crucial with a clear placement of responsibility when it comes to keeping the list updated. In the current version, only the GP can update the list, but when the eSML is implemented nationwide, all prescribers will be able to make changes to the same list. The last physician adding, withdrawing or changing a prescription item will then have to take responsibility for the medication list as a whole. This means they will have to delete prescriptions that are no longer relevant or appropriate, also those prescribed by other doctors. This is currently the case for ordinary e-prescriptions as well: a physician can delete a prescription another physician has written. However, this is often not done: in March 2021, it was estimated that 13% of patients have at least one duplicate prescription in the Norwegian prescription database [47]. These non-current and duplicate prescriptions pose a serious threat to the long-term trustworthiness of the eSML. Not only can these prescriptions lead to patients getting the wrong medicine or dose, but if physicians are reluctant to delete prescriptions issued by other doctors [48] this might with time result in more polypharmacy, inappropriate prescribing and decreased patient safety.

## Conclusions

This study suggests that an eSML reduces the number of discrepancies between the medication lists of the GP, the home care service and the pharmacy for MDD users. Before implementation, the overlap of unique prescription items in the tree lists was 77% in the intervention group; after, it had increased to 94%. For the control group, there was no change. Despite a great improvement, discrepancies were still present, showing that the eSML does not eliminate the need for medication reconciliations. The lessons learned from the MDD patients in our study is that medication reconciliation and medication review must go hand-in-hand to support the construction of the first eSML. Further studies, before the nationwide implementation should focus on how GPs, in collaboration with other health care professionals and the patient, create the first eSML based on the many medication lists that are available today.

## Data Availability

Data are available upon reasonable request to the corresponding author.

## Abbreviations

eSML: Electronically Shared Medication List
MDD: Multidose Drug Dispensing
DID: Difference in Difference
EHR: Electronic Health Record
